# Colon Cancer with* Streptococcus gallolyticus* Aortic Valve Endocarditis: A Missing Link?

**DOI:** 10.1155/2019/4205603

**Published:** 2019-07-03

**Authors:** Chukwunonso Chime, Harish Patel, Kishore Kumar, Ahmed Elwan, Manoj Bhandari, Ariyo Ihimoyan

**Affiliations:** BronxCare Health System, A Clinical Affiliate of Mt Sinai Health Systems and an Academic Affiliate of Icahn School of Medicine, USA

## Abstract

Bacterial endocarditis is commonly encountered in clinical practice. Many bacterial species have been implicated; however,* Streptococcus gallolyticus* species (formerly “*bovis*”) has driven attention given a historical association with colon cancer. Colonoscopy is recommended in an individual with* S. gallolyticus *endocarditis or bacteremia to evaluate the possibility of high-grade adenoma or colon cancer. There has been no firm recommendation for prophylactic antibiotics to prevent bacterial endocarditis for patients undergoing endoscopic procedures and postcolonoscopy bacteremia in an individual with an endoscopic procedure indicated for* S. gallolyticus *bacteremia has not been reported. Studies have been aimed at understanding the association between colon cancer and this bacterial pathogen. There are suggestions that the systemic manifestation of* S. gallolyticus*, a commensal in the colon premalignant cells, may be further predisposed by patient's immunocompromised status. We present a case of the 72-year-old man with the newly diagnosed multiple myeloma presented with aortic valve endocarditis and* S. gallolyticus *bacteremia. Colonoscopy revealed colon cancer and high-grade adenoma; few hours after procedure, he presented with* Streptococcus mitis *bacteremia. In conclusion, our case realigns association of* S. gallolyticus *to colon cancer, especially in an individual with altered immunity, and is novel to demonstrate the rare association of two distinct bacteria of* Streptococcus *species associated with cancer. Preendoscopic antibiotics use, though not standard of care, can be considered in the high-risk individual. Altered immunity can be considered the “missing link” inciting bacteremia in individuals with* S. gallolyticus*-associated colon cancer.

## 1. Introduction

The carriage rate of* Streptococcus gallolyticus* is low in the general population. However, a much higher carriage rate is observed in patients with colon cancer [1].* S. gallolyticus* contributes to 13% of all infective endocarditis [2]. There have been several studies showing a close association between* S. gallolyticus* endocarditis and colon cancer [3], with incidence between 18% and 62% [4]. It has been standard of care to screen the patients with* S. gallolyticus* endocarditis for colon cancer. The pathogenesis of the progression of the colonization to bacteremia is poorly understood. We opine that underlying overt or covert humoral or cellular immune deficiency may be one of the predisposing factors, as we suspect was the case in our index patient, diagnosed with multiple myeloma before clinically apparent infection of endocarditis.

## 2. Case Presentation

A 72-year-old male who recently migrated from Dominican Republic was seen in the ambulatory clinic with few weeks of intermittent fevers and bilateral lower limb edema. His medical comorbid condition is significant for hypertension and spondyloarthropathy. He required hospitalization of the further evaluation of echocardiographic finding of aortic valve vegetation.

Patient's illness started with lower back pain 2 months before presentation, further evaluation anemia, and bone lesion leads to bone marrow biopsy concluding the diagnosis of multiple myeloma. Weight loss of 17 pounds during this period was also associated. Review of system was positive for malaise and constipation. He did not report rectal bleeding, change in stool caliber, or difficulty in swallowing. He had a prior history of remote knee surgery in childhood; only social habit was occasional alcohol use and physical examination was unremarkable except for both diastolic and systolic murmur over the aortic area.

**Figure 1:**
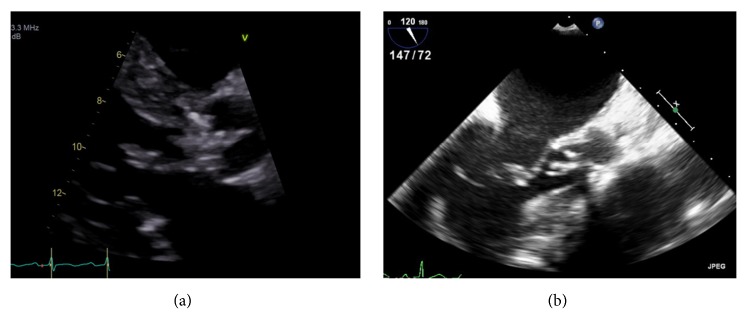
(a) Transthoracic echocardiogram on parasternal long axis view shows independently mobile echo dense structure of 1.61 cm x 0.82 cm on the LVOT side, likely vegetation. (b) Transesophageal echocardiogram mid esophagus long axis view shows the aortic valve with the aortic valve vegetation.

The initial laboratory parameters reveal hemoglobin of 8.5 g/dl and white cell count of 9.2 x I0^9^ cell/dl (83.6% neutrophils); transthoracic echocardiogram showed preserved ejection fraction, moderate aortic stenosis, and regurgitation with thickening of the aortic valve and independently mobile echo dense structure on the left ventricular outflow tract measuring 1.61 × 0.82 centimeters (see Figure 1(a)). Transesophageal echocardiogram showed tricuspid aortic valve with moderate aortic stenosis, moderate aortic regurgitation, and large aortic valve vegetation of 0.45 cm x 1.3 cm attached to right cusp (see Figure 1(b)). Three sets of blood culture reported growth of* S. gallolyticus* from both aerobic and anaerobic bottles with culture and sensitivity depicting sensitivity to Vancomycin and Ceftriaxone. Infectious disease consultation recommended both intravenous (IV) antimicrobials for six weeks.

**Figure 2:**
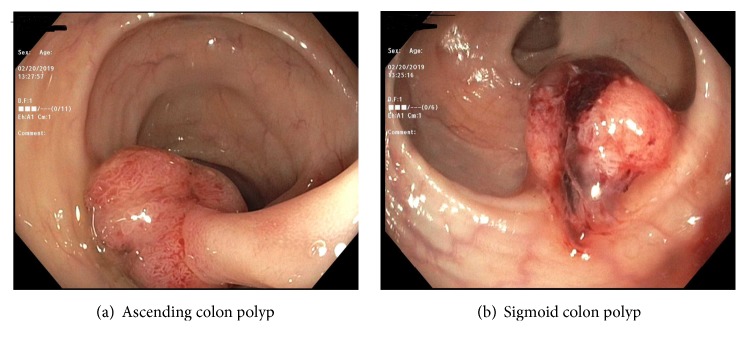
High-definition colonoscopy images showing pedunculated polyp in the ascending colon and a nonpedunculated polyp in the sigmoid colon.

He was started on intravenous Vancomycin and Ceftriaxone and transferred to a higher tertiary center for further surgical management, where he had aortic valve replacement with an Edwards 23 mm bioprosthetic valve. He had an uneventful recovery after completion of a six-week course of antibiotics (Ceftriaxone and Vancomycin) and was referred to the gastroenterology clinic for a screening colonoscopy. The colonoscopy revealed a 35 mm polyp in the sigmoid colon and another 30 mm polyp in the descending colon (see Figures 2(a) and 2(b)), both removed with hot snare polypectomy. He presented the next day with the high-grade fever with blood cultures positive for* Streptococcus mitis*; repeat echocardiogram showed no vegetation on the native or bioprosthetic valve. He was treated with a repeat course of antibiotics. Pathology of the sigmoid colon polyp was consistent with invasive adenocarcinoma arising from tubular adenoma and the ascending colon polyp revealed tubular adenoma with high-grade dysplasia. After imaging studies, he staged to colon cancer AJCC Stage 1 (Duke's stage A). Subsequently, he underwent a right hemicolectomy with clean resection margins and no evidence of metastasis and made a remarkable recovery. For the management of multiple myeloma, he is being planned for Bortezomib-based chemotherapy followed by stem cell transplant.

## 3. Discussion

Genetic and environmental factors play a role in the pathogenesis of colon cancer; environmental factors include viruses, diet, and gut microbiota [5]. Gut dysbiosis can be one of the factors contributing to pathogenesis colorectal cancer [6]. Colon cancer has been linked with bacterial species including* Streptococcus gallolyticus, Enterococcus faecalis, Bacteroides fragilis, Escherichia coli, Clostridium septicum, *and* Fusobacterium *spp. [6]. Historically,* Mc Coy et* al. reported first case of cooccurrence of colon cancer with bacterial endocarditis [7]. The first association of the colon cancer and* S. gallolyticus *surfaced in 1974; Klein et al. demonstrated higher rates of* S. gallolyticus *in the stool culture of the individuals with colon cancer [8].

*Streptococcus gallolyticus*, previously* Streptococcus bovis* biotype I, is a group D streptococcus responsible for infective endocarditis (IE) and bacteremia, especially in the older age groups [9]. For decades, we have known about the strong relationship between colorectal cancer and* S. gallolyticus* (formerly* bovis*). Recent studies have demonstrated that colon cancer development is promoted actively by* S. gallolyticus* through the B-catenin pathway mediated cell proliferation [10].* S. gallolyticus *with its ability to express pili involved in mucin (Pil3) and collagen (Pil1) binding can attach to infection sites of the colon and cardiac valves, respectively [11, 12].

Antibody response to the pilus proteins of* S. gallolyticus *has been associated with colon cancer, especially in individuals below 65 years of age [13]. Follow-up of a colon cancer patient in Spain revealed that antibody response to pilus protein Gallo 2039, Gallo2178, and Gallo 2179 has been associated with an increased risk of colon cancer [13]. Antibody response to* S. gallolyticus* is mounted prior to emergence of malignancy in the colonic tissue [14].

Premalignant colon lesions likely enable colonization of the colon by* S. gallolyticus*, as the bacteria survive for long periods given a competitive advantage that the premalignant microenvironment provides [15]. In most cases, the presence of advanced adenoma or invasive cancer with* S. gallolyticus *bacterial colonization precedes the diagnosis of endocarditis. In our index patient, colonoscopy was performed three months after initial diagnosis of aortic valve endocarditis and findings revealed sigmoid colon invasive adenocarcinoma with synchronous high-grade adenoma in descending colon revealing carcinoma in situ. It has been long known that these bacteria translocate from the gut to the systemic circulation through portals of entry that these premalignant lesions provide [16]. The pertinent question is “what triggers the development of clinically apparent bacteremia and endocarditis?”

*Abeni *et al. described a case of* S. gallolyticus (bovis)* related infective endocarditis that manifested in context to neutropenia in the setting of the chemotherapy for colon cancer [3]. This is further backed up by data reporting silent infection in a majority of patients with colon premalignant and malignant lesion, which becomes clinically apparent with the development of immune system disorders [17]. The bacteria likely overwhelm the immune system to trigger septicemia, but we propose that, in a subset of cases, some overt or occult humoral and/or cellular immune deficiency likely contributes to the susceptibility of the host to progression to systemic manifestations like endocarditis. Our index patient developed endocarditis after he was diagnosed with multiple myeloma, which likely predisposed him to clinical manifestation of bacteremia and endocarditis.

Cancer-related dysbiosis can lead to an increase in oral* Streptococcus mitis *colony-forming unit (CFU) [18].* S. Mitis* has been associated with severe infectious outcomes in the patient with cancer [19]. There has been no prior correlation of* Streptococcus mitis *with colon cancer. We opine that the* Streptococcus mitis *bacteremia may be incited by the altered immunity in conjunction with the colon malignancy. The bacteremia by two different streptococci species spaced out in time in presence of colon cancer has never been reported.

Colonoscopy has less than 5% risk of bacteremia and* Staphylococcus epidermidis *is the most common isolate [20]. The rates of the bacteremia during polypectomy are comparable to other gastrointestinal procedures [21]. In view of the low risk of bacteremia, the routine use of antibiotics is not recommended in gastrointestinal procedures [22], though in the high-risk case, we propose that antibiotics for infective endocarditis can be considered.

The management for endocarditis entails intravenous antibiotics and surgical intervention if needed. In patients with* S. gallolyticus *as the causative organism, colonoscopy is recommended to further evaluate for premalignant and malignant colon lesions. We propose that patients should also be worked up for any overt or occult immunodeficiency states that may have incited a contributory role to the progression to clinically overt bacteremia.

## 4. Conclusion

Historically, there has been a close association between infective endocarditis caused by* S. gallolyticus* and colon cancer. Patients with* S. gallolyticus* bacteremia do need colorectal cancer evaluation.* S. gallolyticus* bacteremia can be incited by immune compromised status in the individual with preexisting colon cancer, as reported in the index case. Periendoscopic antibiotics prophylaxis, though not standard of care, should be considered in the high risk individuals. Further consideration should be given to work up for possible underlying immune compromise status in patients with clinically apparent bacteremia, as that could be a missing link to the current timing of systemic manifestation.
